# Long intergenic non-protein-coding RNA 01446 facilitates the proliferation and metastasis of gastric cancer cells through interacting with the histone lysine-specific demethylase LSD1

**DOI:** 10.1038/s41419-020-2729-0

**Published:** 2020-07-10

**Authors:** Yifan Lian, Changsheng Yan, Yikai Lian, Renzhi Yang, Qiongyun Chen, Dan Ma, Weibin Lian, Jingjing Liu, Chengyan Luo, Jianlin Ren, Hongzhi Xu

**Affiliations:** 1 Department of Gastroenterology, Zhongshan Hospital, Xiamen University, Xiamen, Fujian PR China; 2 Institute for Microbial Ecology, School of medicine, Xiamen University, Xiamen, Fujian PR China; 3 Department of Obstetrics and Gynecology, The First Affiliated Hospital of Xiamen University, Xiamen, Fujian PR China; 4 Department of Breast Surgery, Quanzhou First Hospital Affiliated to Fujian Medical University, Quanzhou, Fujian PR China; 5 Department of Gynecology, The First Affiliated Hospital of Nanjing Medical University, Nanjing, Jiangsu PR China

**Keywords:** RNA, Cancer epidemiology

## Abstract

Growing evidences illustrated that long non-coding RNAs (lncRNAs) exhibited widespread effects on the progression of human cancers via various mechanisms. Long intergenic non-protein-coding RNA 01446 (LINC01446), a 3484-bp ncRNA, is known to locate at chromosome 7p12.1. However, its biological functions and specific action mechanism in gastric cancer (GC) are still unclear. In our study, LINC01446 was proved to be markedly upregulated in GC tissues relative to the normal tissues, and positively correlated with the poor survival of GC patients. The multivariate Cox regression model showed that LINC01446 functioned as an independent prognostic factor for the survival of GC patients. Functionally, LINC01446 facilitated the proliferation and metastasis of GC cells. Moreover, RNA-seq analysis demonstrated that LINC01446 knockdown primarily regulated the genes relating to the growth and migration of GC. Mechanistically, LINC01446 could widely interact with histone lysine-specific demethylase LSD1 and recruit LSD1 to the Ras-related dexamethasone-induced 1 (RASD1) promoter, thereby suppressing RASD1 transcription. Overall, these findings suggest that LINC01446/LSD1/RASD1 regulatory axis may provide bona fide targets for anti-GC therapies.

## Introduction

Gastric cancer (GC), a main factor in causing cancer-related mortalities all over the world, is one of the most common gastrointestinal malignancies in China^1–3^. Until 2015, GC was considered as the second most common cancer following lung cancer in China based on the latest cancer statistic data^1^. Although the diagnosis and therapies for GC have greatly improved, there are still high recurrence and metastasis rates^4^. Moreover, these improvements are often limited because the related molecular mechanisms underlying the occurrence and development of GC remain unclear^5^. Thus, it is critical to understand the potential action mechanism of GC for discovering novel therapies for GC.

Transcriptome studies indicated that more than 70% of human genomes could be transcribed to RNAs, and most of them were non-coding^6^. In the recent years, some studies on GC action mechanism revealed that long non-coding RNAs (lncRNAs), the epigenetic regulators at transcriptional or posttranscriptional levels, exhibited crucial effects on the proliferation, apoptosis, and metastasis of GC cells, and drug-resistance^7–9^. In addition, other studies indicated that lncRNAs could also serve as potential therapeutic targets for GC, and might be diagnostic and prognostic biomarkers for GC^7,9,10^. Specifically, low expression of LINC01133 suppressed the proliferation and metastasis of GC cells via regulating APC expressions and Wnt/β-catenin signals^11^; lncRNA GMAN was related to the metastasis of GC and could promote ephrin A1 translation via competitively binding to GMAN-AS^12^; and lncRNA HOXC-AS3 could promote the occurrence of GC via regulating HDAC5 at transcriptional level^13^. These studies together indicate that as an important kind of non-coding RNAs, lncRNAs exhibit wide effects on regulating the occurrence and development of GC. However, the related functions and specific regulatory mechanism of lncRNAs in GC need further studies to provide potential therapeutic targets for GC.

LINC01446 has been reported in glioblastoma^14^. However, it is unclear whether LINC01446 exerts similar function in GC tumorigenesis. In our study, LINC01446 expression in GC was detected to understand the function and potential mechanism of LINC01446 regulating the proliferation and metastasis of GC cells. The results demonstrated that the increased LINC01446 expression in GC was related to the metastasis of GC and shorter survival time of GC patients. It was also found that LINC01446 knockdown obviously reduced the proliferation and migration of GC cells. Moreover, LINC01446 was confirmed to downregulate the Ras-related dexamethasone-induced 1 (RASD1) expression at transcriptional level through recruiting the activated histone lysine-specific demethylase LSD1 to the promoter area of RASD1, thereby accelerating the proliferation and metastasis of GC cells.

## Results

### LINC01446 is upregulated in GC and related to poor prognosis

**Fig. 1:**
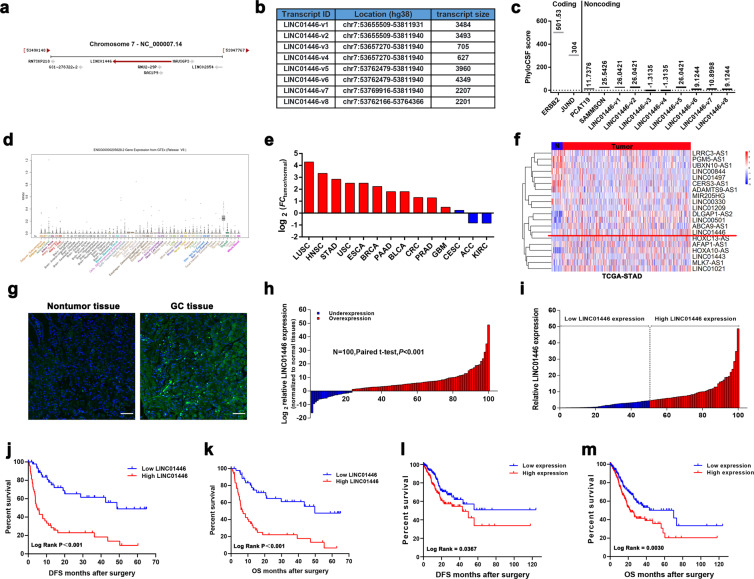
LINC01446 expression is upregulated in GC tissues and related to the poor prognosis of GC patients. **a** Schematic annotation for LINC01446 genomic locus. Arrows represented the orientation of transcript. **b** The list of the human genomic location from lncRNA LINC01446 family. **c** The maximum cerebrospinal fluid scores of LINC01446 and other known coding and non-coding RNAs were assessed by using PhyloCSF analysis. **d** LINC01446 expression profile in normal tissues was obtained from UCSC Genome Browser database. **e** Fold change (FC) of LINC01446 expression (tumor/adjacent normal tissues) in multiple types of cancers from TCGA. **f** LINC01446 expression in GC from TCGA database. **g** Expression of LINC01446 was detected by using FISH. The green represented positive signal. Scale bar: 80 μm. **h** Analysis for LINC01446 mRNA expressions in 100 matched GC and normal tissues. **i** GC patients were assigned into two groups following the LINC01446 expression. **j, k** Kaplan–Meier analysis for disease-free survival and overall survival following the LINC01446 mRNA expression level. **l, m** Kaplan–Meier analysis for disease-free survival and overall survival following the LINC01446 mRNA expression level from TCGA database. **h**, **i** Student’s *t* test. **j**–**m** Log-rank test. *P < 0.05. n.s. not significant.

As shown at https://www.ncbi.nlm.nih.gov/gene/?term=LINC01446, human LINC01446 is located at chromosome 7 and includes eight transcripts. LINC01446-v1, an isoform of LINC01446, is consisted of six exons and highly conservative in mammals (Fig. 1a, b). Subsequently, rapid amplification of cDNA ends was performed to identify the full sequence of LINC01446 in SGC7901 cells according to the sequence archived in the RefSeq database of NCBI (full exact length 3484 bp, as shown in Fig. S1a). The analysis for coding potential obviously demonstrated that all isoforms of LINC01446 might lack the protein-coding ability (Fig. 1c). The GTEx data available at https://www.gtexportal.org/ illustrated that there was a low expression of LINC01446 in normal tissues (Figs. 1d and S1b). Moreover, TCGA pan-cancer analysis revealed that LINC01446 was most significantly upregulated in GC among all types of cancers (Fig. 1e, f). Interestingly, according to the analysis of TCGA data, the expression level of LINC01446 was significantly higher in both of the intestinal type and mixed type of GC compared with that of normal tissues (Fig. S1c). More importantly, the in situ hybridization assay demonstrated that LINC01446 was expressed in GC tissues, but not in the normal tissues (Fig. 1g). Correspondingly, subsequent quantitative real-time PCR (qRT-PCR) analysis in our study revealed that LINC01446 expression was much higher in GC tissues than in the normal tissues (Fig. 1h).

To further assess the key roles LINC01446 played, we investigated the relationship between the clinicopathological characteristic of GC patients and LINC01446 expression. At first, these 100 patients were assigned into low (*n* = 50) and high LINC01446 expression (*n* = 50) groups based on the median value (Fig. 1i). The results showed that the high-expressed LINC01446 in GC patients was positively related to the advanced TNM and distal metastasis (Table 1). Moreover, Kaplan–Meier analysis demonstrated that the high-expressed LINC01446 was also related to the reduced overall survival (OS) of patients (median DFS times: 4.69 vs. 48.72 months, *P* < 0.001; median OS times: 6.69 vs. 49.72 months, *P* < 0.001) (Fig. 1j, k), which was consistent with the results from the Kaplan–Meier analysis for TCGA-STAD database (median OS times: 22.03 vs. 70.00 months, *P* = 0.0065) (Fig. 1l, m). Moreover, Multivariate Cox analysis further demonstrated that the high-expressed LINC01446 was independently related to the reduced OS (DFS, hazard ratio = 4.928; 95% CI: 2.671–9.090; *P* < 0.001; OS, hazard ratio = 4.446; 95% CI: 2.409–8.206; *P* < 0.001) (Table 2). In summary, our findings together indicated that LINC01446 overexpression is an independent prognostic indicator for GC patients and exhibits key effects on GC progression.

**Table 1:** Correlation between LINC01446 expression and clinicopathologic characteristics of patients with GC (*n* = 100).

Characteristics	LINC01446		*P*
	Low no. cases (*n* = 50)	High no. cases (*n* = 50)	Chi-squared test, *P* value
Age (years)			0.509
≤60	16	13	
>60	34	37	
Gender			0.175
Male	26	23	
Female	24	27	
Lauren classification			0.657
Diffuse type	21	23	
Intestinal type	25	21	
Mixed type	4	6	
Stage			**0.036**
I	14	5	
II	9	6	
III	16	17	
IV	11	22	
Distal metastasis			**0.019**
M0	39	28	
M1	11	22	

Significant *P* values are shown in bold font.

**Table 2:** Univariate and multivariate Cox regression analysis of LINC01446 and survival in patients with GC.

Variables	Univariate analysis			Multivariate analysis		
	*P* value	HR	95% CI	*P* value	HR	95% CI
OS						
Age (≤60, >60)	0.995	1.000	0.979–1.022	0.960	0.999	0.977–1.022
Gender (male, female)	0.125	0.636	0.357–1.134	0.441	0.784	0.421–1.457
Lauren classification	0.154	0.720	0.459–1.131	0.021	0.576	0.361–0.919
TNM stage (I/II, III/IV)	**<0.001**	2.610	1.843–3.696	**<0.001**	2.547	1.775–3.655
Tumor invasion depth (T1/T2, T3/T4)	0.064	1.283	0.985–1.671	0.440	0.895	0.677–1.185
Lymph node metastasis (N0, N1)	0.003	1.418	1.125–1.786	0.760	0.952	0.693–1.308
Distant metastasis (yes, no)	**<0.001**	4.400	2.432–7.959	0.991	1.007	0.297–3.413
LINC01446 expression	**<0.001**	5.730	3.106–10.568	**<0.001**	4.446	2.409–8.206
DFS						
Age (≤60, >60)	0.993	1.000	0.979–1.022	0.946	1.001	0.978–1.024
Gender (male, female)	0.122	0.626	0.351–1.116	0.371	0.752	0.403–1.403
Lauren classification	0.153	0.721	0.460–1.028	0.020	0.576	0.362–0.917
TNM stage (I/II, III/IV)	**<0.001**	2.594	1.834–3.670	**<0.001**	2.566	1.786–3.687
Tumor invasion depth (T1/T2, T3/T4)	0.069	1.275	0.981–1.657	0.371	0.881	0.667–1.163
Lymph node metastasis (N0, N1)	0.002	1.433	1.137–1.806	0.983	0.996	0.698–1.421
Distant metastasis (yes, no)	**<0.001**	4.346	2.405–7.851	0.845	1.113	0.379–3.267
LINC01446 expression	**<0.001**	6.126	3.324–11.289	**<0.001**	4.928	2.671–9.090

HR > 1, risk for death increased; HR < 1, risk for death reduced. Significant *P* values are shown in bold font.

*OS* overall survival, *DFS* disease-free survival, *TNM* tumor node metastasis, *HR* hazard ratio, *CI* confidence interval.

### LINC01446 facilitates the proliferation and metastasis of GC cells in vitro

**Fig. 2:**
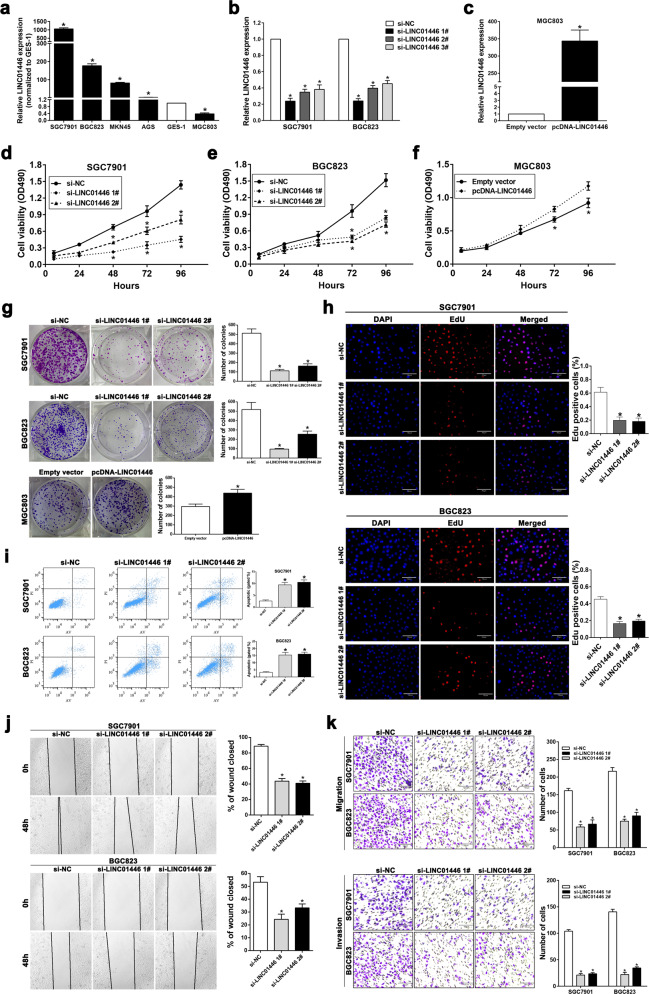
LINC01446 facilitates the proliferation and migration of GC cells in vitro. **a** LINC01446 mRNA expressions in five GC and GES-1 cells were measured. **b, c** LINC01446 knockdown or overexpression efficiencies in GC cells were assessed. **d, e** MTT assay for the si-LINC01446- and si-NC-transfected SGC7901 and BGC823 cells. **f** MTT assays were conducted in the LINC01446-overexpressed MGC803 cells. **g** The proliferation of the transfected SGC7901 and BGC823 cells with si-LINC01446 or the MGC803 cells overexpressed by plasmid were detected by using colony formation assay. **h** Representative images (left) and quantification (right) for EdU immunofluorescence staining in the si-LINC01446- and si-NC-transfected SGC7901 and BGC823 cells. Scale bar: 70 μm. **i** Apoptosis in the si-LINC01446- and si-NC-transfected SGC7901 and BGC823 cells was determined using flow cytometry. **j** The migration of the LINC01446-silenced and control cells was investigated using wound-healing assay. **k** The migratory and invasive abilities of LINC01446-silenced and control cells were detected using transwell assay. Scale bar: 120 μm. Data were shown as mean ± SD, *n* = 3. Student’s *t* test. *P < 0.05. n.s. not significant.

**Fig. 3:**
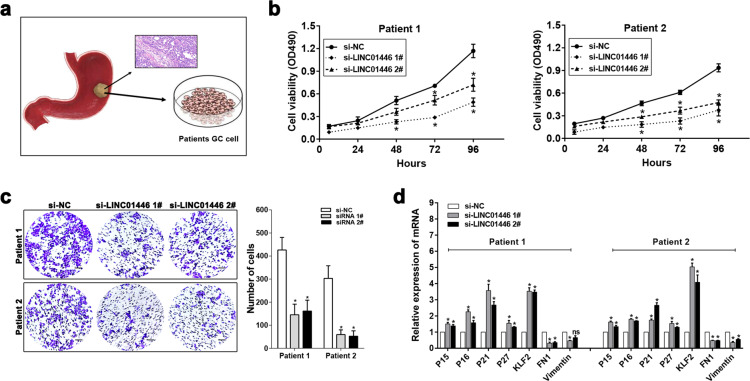
Primary GC cells confirmed the anticancer effect by LINC01446 silencing. **a** GC tissues from two patients were obtained from the hospital, we isolated primary GC cells by collagenase digestion. **b** MTT assays for the si-LINC01446- or si-NC-transfected primary GC cells from patient 1 and patient 2. **c** The migratory abilities of LINC01446-silenced and control cells were detected using transwell assay. Scale bar: 120 μm. **d** P15, P16, P21, p27, KLF2, FN1, and Vimentin mRNA expression levels were determined by qRT-PCR following LINC01446 knockdown. Data were shown as mean ± SD, *n* = 3. Student’s *t* test, *P < 0.05. n.s. not significant.

Given that there was a strong relationship between LINC01446 expression in GC and worse prognosis of GC patients, we further explored whether LINC01446 facilitates the proliferation, invasion, and migration of GC cells. Following detecting LINC01446 expressions in five GC cells and GES-1 cells, we interfered LINC01446 expressions in the SGC7901 and BGC823 cells that had high LINC01446 expression using si-LINC01446, and overexpressed LINC01446 in the MGC803 cells that had low LINC01446 expression (Fig. 2a–c). As showed in Fig. 2d–g, MTT and colony formation assays showed that LINC01446 interference significantly disrupted the proliferation and colony formation of SGC7901/BGC823 cells. On the contrary, LINC01446 overexpression facilitated the cell proliferation in vitro. Similarly, ethynyldeoxyuridine (EdU)/DAPI immunostaining supported this finding (Figs. 2h and S2a). In addition, apoptosis was identified as a key factor contributing to GC cell growth, so we conducted flow cytometry to detect the apoptosis level. The results demonstrated that LINC01446 knockdown by si-LINC01446 significantly increased the percentage of apoptotic cells (Fig. 2i), whereas excessive LINC01446 weakened the percentage of apoptotic cells (Fig. S2b). TUNNEL staining analysis verified the antiapoptotic character of LINC01446 (Fig. S2c). Moreover, wound-healing and transwell assays illustrated that LINC01446 interference markedly suppressed the invasiveness and migration of SGC7901 and BGC823 cells (Fig. 2j, k); in contras, the invasiveness and migration were observably increased depending on the overexpression of LINC01446 in MGC803 cells (Fig. S2d, e). To further evaluate the anticancer effect of si-LINC01446 and confirm our observation in GC cell lines, we interfered LINC01446 in primary GC cells that were isolated from tumor tissues from two GC patients (Fig. 3a). The clinical information about these two patients were listed in Table S4. As expected, the results of MTT and transwell assays demonstrated that the viability and migration of primary GC cells obviously decreased following LINC01446 knockdown (Fig. 3b, c). Correspondingly, the mRNA levels of P15, P16, P21, P27, KLF2 also markedly increased when LINC011446 was interfered (Fig. 3d). Collectively, our data indicated that si-LINC01446 exhibited oncogenic effects on markedly suppressing the proliferation and metastasis of GC cells and the proper regulation for its activity might be a promising strategy for GC treatment.

### LINC01446 facilitates the tumor growth and metastasis of GC in vivo

**Fig. 4:**
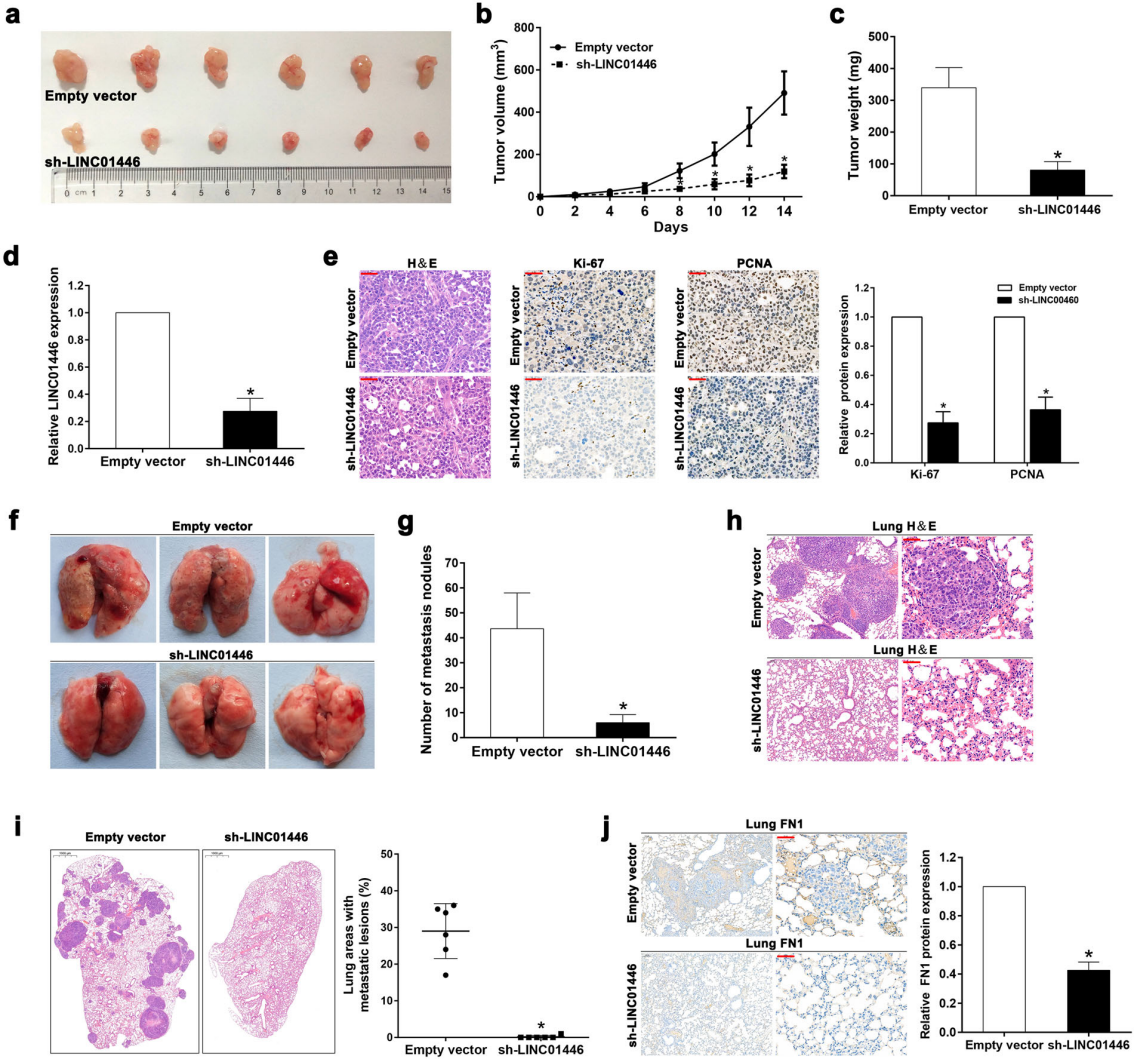
Effects of LINC01446 on the tumor growth and metastasis of GC in vivo. **a** The transfected SGC7901 and BGC823 cells with empty vector or sh-LINC00707 were inoculated into nude mice. **b** Tumor volume was determined after injecting every 2 days. **c** Tumors were weighted following removing. **d** The mRNA expressions of LINC01446 in xenograft tumors were measured. **e** Tumor sections were stained by H&E and IHC using anti-Ki-67 and anti-PCNA antibodies. The representative staining images were shown. Scale bar: 50 μm. **f** The representative images for the entire lung of mice from each group were shown. **g** The numbers of metastasis nodules on the surface of lungs were counted. **h** Lung sections were stained by H&E. Scale bar: 50 μm. **i** The percentage of lung areas with metastatic lesions was calculated from all mice in each group. Scale bar: 1000 μm. **j** Lung sections were stained by IHC with anti-FN1 antibody. The representative staining images were shown. Scale bar: 50 μm. Data were shown as mean ± SD. Student’s *t* test. *P < 0.05. n.s. not significant.

To further evaluate the effects LINC01446 on the tumor growth of GC, the stably transfected SGC7901 cells with sh-LINC01446 or empty vectors were subcutaneously injected into BALB/c mice, and the tumor growth was measured. The results showed that the tumors were smaller following LINC01446 knockdown than those without LINC01446 knockdown (Fig. 4a). Accordingly, the volume and weight of tumors also obviously decreased in sh-LINC01446 group relative to the empty vector group (Fig. 4b, c). qRT-PCR result demonstrated that the LINC01446 mRNA expression in the tumor tissues with sh-LINC01446 cell transfection was lower than that with control cell transfection (Fig. 4d). Moreover, immunohistochemistry (IHC) showed that the expressions of proliferation marker Ki-67 and proliferating cell nuclear antigen (PCNA) significantly reduced in the sh-LINC01446-transfected SGC7901 cells relative to the control cells (Fig. 4e).

To further explore the roles LINC01446 plays in the metastasis of GC in vivo, an in vivo mouse model with lung metastasis was constructed. As requested, the stably transfected SGC7901 cells with sh-LINC01446 or empty vectors were injected into the tail veins of six mice, then the metastatic nodules on the surfaces of their lungs were calculated after 6 weeks. The results demonstrated that the LINC01446 interference markedly reduced the numbers of the metastatic nodules (Fig. 4f, g). This finding was further verified by the subsequent examination for the whole lung and the H&E staining for lung tissues (Fig. 4h). Next, the metastatic lesions in lung sections were also detected by H&E staining, and the result indicated that the percentage of the lung areas with metastatic lesions was much lower in the sh-LINC01446-transfected group than in the empty vector-transfected group (Fig. 4i). In addition, cell adhesion marker fibronectin1 (FN1) expression was also detected using IHC staining, demonstrating that the FN1 expression obviously decreased in the lungs transfected by sh-LINC01446 cells (Fig. 4j).

In summary, all the above data confirmed that the knockdown of LINC01446 could markedly suppress the tumor growth and metastasis of GC, which further validated the oncogenic roles LINC01446 played in GC.

### LINC01446 interacts with the histone lysine-specific demethylase LSD1

**Fig. 5:**
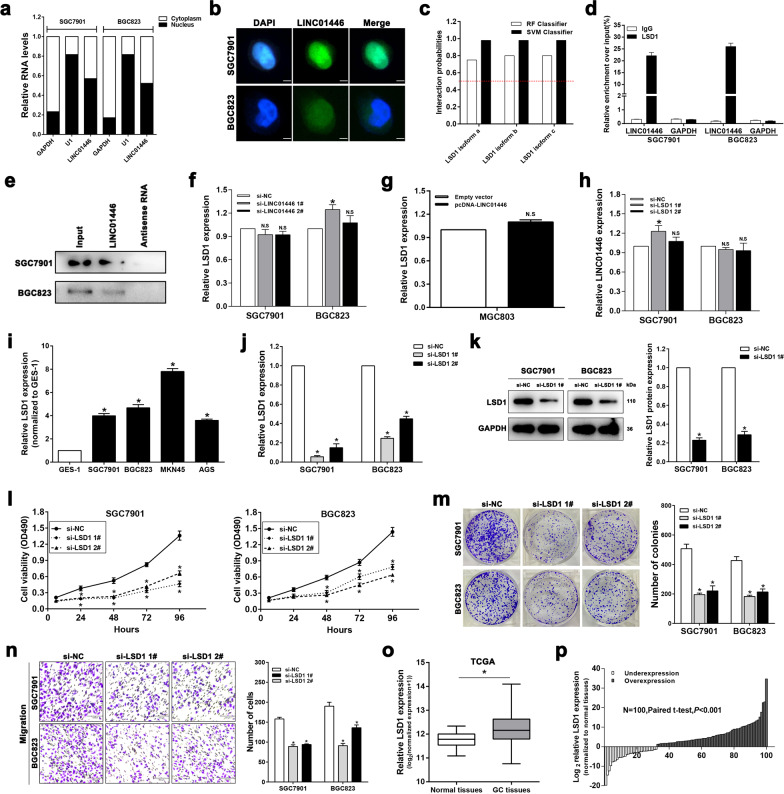
LSD1 acts as an oncogene in interacting with LINC01446 in GC. **a** The levels of U1, GAPDH, and LINC01446 in nuclear and cytoplasmic fractions were detected using qRT-PCR. **b** RNA FISH analysis for the LINC01446 levels in SGC7901 and BGC823 cells. Nuclei were stained using DAPI. Scale bar: 5 μm. **c** The relationship between LINC01446 and LSD1 was predicted using RNA-Protein interaction prediction (RPISeq) website, and the prediction probability more than 0.5 represented that there was an interaction probability between the corresponding RNA and protein. **d** RIP was conducted to detect the LINC01446 mRNA level in the co-precipitated RNA. GAPDH was employed as a negative control. **e** LINC01446 and antisense RNA were co-incubated with cell extracts, and LSD1 proteins were measured. **f**, **g** The mRNA expressions of LSD1 in GC cells after LINC01446 knockdown or overexpression were measured. **h** The levels of LINC01446 in GC cells were detected after LSD1 knockdown. **i** The mRNA expressions of LSD1 in five GC and GES-1 cells were detected. **j**, **k** The LSD1 knockdown efficiency in GC cells was evaluated. **l** MTT assays for the si-LSD1- or si-NC-transfected SGC7901 and BGC823 cells. **m** Colony formation assay was conducted to measure the proliferation of the si-LSD1-transfected SGC7901 and BGC823 cells. **n** Transwell assay was carried out to determine the migratory and invasive abilities of the LSD1-silenced and control cells. Scale bar: 120 μm. **o** LSD1 expression in GC cells from TCGA database. **p** Analysis for LSD1 levels in 100 matched GC and adjacent normal tissues using qRT-PCR. Data were shown as mean ± SD, *n* = 3. Student’s *t* test. *P < 0.05. n.s. not significant.

Growing evidences indicated that different biological roles lncRNAs played greatly depended on their subcellular localizations^15,16^. As Fig. 5a, b showed, LINC01446 expression in the nucleus was more than that in the cytosol of SGC7901 and BGC823 cells, suggesting that LINC01446 may transcriptionally exhibit main regulatory effects. LSD1, an epigenetic, can specifically promote H3K4me2 demethylations, thereby regulating gene transcription. Therefore, we subsequently employed bioinformatics analysis to investigate whether LINC01446 binds to LSD1. The results showed that RF and SVM Classifier scores were more than 0.5 (Fig. 5c), suggesting that LINC01446 is likely to bind to LSD1. To verify this finding, RNA immunoprecipitation (RIP) assay was conducted and showed that LINC01446 could highly bind to LSD1 in SGC7901 and BGC823 cells (Fig. 5d). Next, RNA pull-down assay was also conducted by using LSD1 antibody to further confirm the relationship between LINC01446 and LSD1. The results demonstrated that the labeled LINC01446 RNA, but an antisense LINC01446, specifically retrieved LSD1 from both SCG7901 and BGC823 cell extracts (Fig. 5e). Furthermore, qRT-PCR analysis demonstrated that there were no changes in LSD1 expressions following the knockdown or overexpression of LINC01446 (Fig. 5f, g), and no change in LINC01446 expression was observed after the knockdown of LSD1 (Fig. 5h). These data together indicate that LINC01446 binds to LSD1 but has no effects on LSD1 expression, suggesting that LINC01446 may regulate the proliferation and metastasis of GC in transcriptional level through its physical interaction with LSD1.

### LSD1 acts as an oncogene in facilitating the proliferation and migration of GC cells

LSD1 was also proved to be associated with the poor prognoses of a variety of tumors^17,18^. Our data demonstrated that the LINC01446 expression was higher in GC cells relative to GES-1 cells (Fig. 5i). To further explore the roles LSD1 plays in the progression of GC, we performed LSD1 knockdown by transfecting si-LSD1 into SGC7901 and BGC823 cells, and then their knockdown efficiencies were evaluated using qRT-PCR and western blotting (Fig. 5j, k). Next, MTT and colony formation assays were carried out to evaluate the roles LSD1 played in the proliferation of GC cells. As expected, LSD1 knockdown significantly suppressed the proliferation and colony formation of SGC7901 and BGC823 cells (Fig. 5l, m). Subsequent transwell assay also showed that LINC01446 knockdown markedly reduced GC cell migration (Fig. 5n). Moreover, further RNA sequencing analysis for TCGA stomach and normal tissues illustrated that LSD1 expression markedly enhanced in GC tissues relative to the normal tissues (Fig. 5o). To further confirm this result, the LSD1 expression in 100 matched GC and normal tissues used in Fig. 1h was measured, and the result indicated that LSD1 was overexpressed in GC tissues (Fig. 5p). All the findings demonstrated that LSD1 could facilitate the proliferation and migration of GC cells, which suggests that LSD1 functions as an important oncogene for GC.

### LINC01446 epigenetically suppresses RASD1 transcription by binding to LSD1

**Fig. 6:**
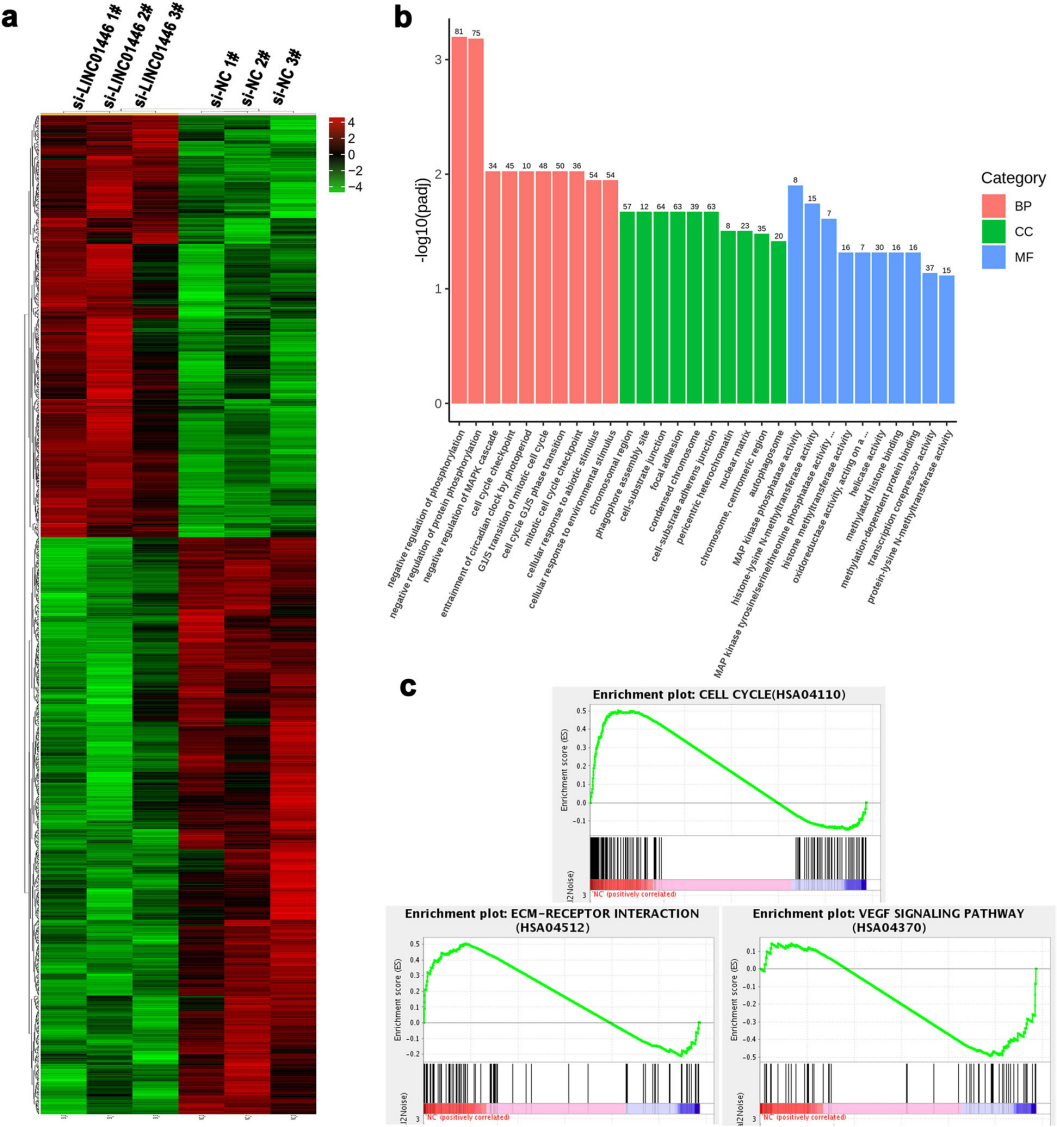
RNA-seq analysis for SGC7901 cells after LINC01446 knockdown. **a** Mean-centered, hierarchical clustering of 1700 altered genes (FC > 1) in si-NC- and si-LINC01446-treated cells in triplicate. **b** GO analysis for all altered genes. **c** GSEA showed that the proliferation and metastasis-related biological functions were enriched in responses to high LINC01446 expression in SGC7901 cells based on the RNA-seq analysis after LINC01446 knockdown.

**Fig. 7:**
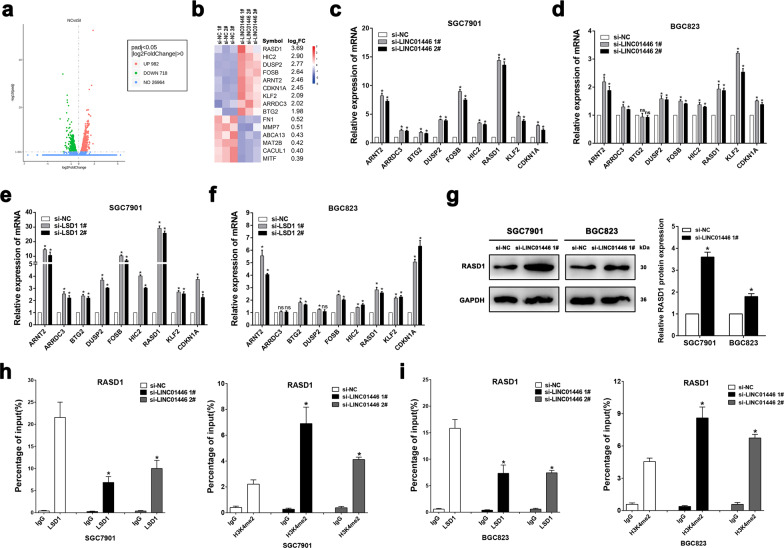
LINC01446 recruits LSD1 to RASD1 promoter and suppresses its transcription. **a** Volcano plot illustrated 294 differentially expressed genes between the si-LINC01446-treated and control SGC7901 cells (FC ≥ 2, *P* < 0.05). **b** Heatmap for the altered genes between the si-LINC01446-treated and control SGC7901 cells. **c**, **d** The mRNA expressions of altered genes were selectively verified after LINC01446 knockdown. **e**, **f** The mRNA expressions of altered genes were selectively verified using qRT-PCR after LSD1 knockdown. **g** The RASD1 expression in SGC7901 and BGC823 cells was analyzed after LINC01446 interference. **h**, **i** The enrichments of LSD1 and demethylated H3K4me2 at the promoter region of RASD1 were assessed using ChIP after LINC01446 knockdown. Data were shown as mean ± SD, *n* = 3. Student’s *t* test. *P < 0.05. n.s. not significant.

To further explore the potential action mechanism of LINC01446 promoting tumorigenesis, the gene expression profiles for RNA sequencing in the LINC01446-knockdown SGC7901 cells and control cells were analyzed by using gene ontology (GO) analysis and gene set enrichment analysis (GSEA) (Fig. 6a). The GO analysis demonstrated that the most significant biological processes included cell cycle, cell adhesion, and cell junction (Fig. 6b). The GSEA illustrated that gene sets were markedly associated with the proliferation and metastasis of tumor cells (such as cell cycle, ECM-receptor interaction, and VEGF signaling pathways) (Figs. 6c and S3). In addition, 1700 genes were differentially expressed (upregulated and downregulated genes) in SGC7901 cells after LINC01446 knockdown (fold change >1) (Fig. 7a). Next, we continued to investigate the specific LINC01446 target that leads to the observed GC cell phenotypes. As expected, several genes associated with the proliferation and metastasis of GC cells were screened and identified by qRT-PCR analysis after LINC01446 and LSD1 knockdowns (Fig. 7b–f). Among these altered genes, RASD1 has been identified as a novel tumor suppressor that regulates the proliferation, apoptosis, and metastasis of cancer cells^19,20^. Therefore, the RASD1 was chosen for the subsequent analyses. Western blot analysis demonstrated that LINC01446 knockdown significantly upregulated RASD1 in both SGC7901 and BGC823 cells (Fig. 7g). Next, two pairs of primers (2000 bp) across the promoter region of RASD1 were designed to perform the chromatin immunoprecipitation (ChIP) assay and investigate the related regulatory mechanism. Our results demonstrated that LSD1 directly bound to the RASD1 promoter region-mediated demethylated H3K4, while LINC01446 interference reduced this bind and promoted the modification in GC cells (Fig. 7h, i). Taken together, our data indicated that RASD1 might be a downstream target gene of LINC01446, and LINC01446 could recruit LSD1 into RASD1 promoter, thereby resulting in epigenetic suppression through demethylated H3K4me2.

### LINC01446 plays oncogenic roles through partly suppressing RASD1 expression

**Fig. 8:**
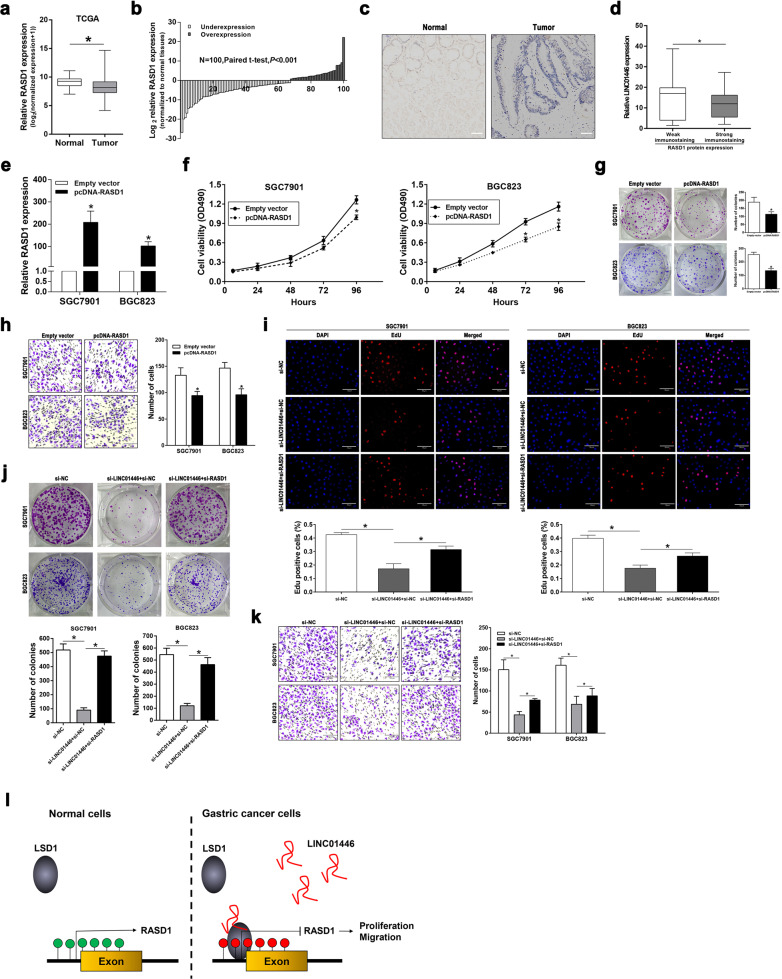
LINC01446 acts as an oncogene by suppressing RASD1 expression in GC cells. **a** RASD1 expression in GC from TCGA database. Wilcoxon rank-sum test was adopted. **b** Analysis for RASD1 levels in 100 matched GC and normal tissues. Student’s *t* test was adopted. **c** IHC staining was employed to measure the RASD1 expression in GC and normal tissues. Spearman’s rank correlation was adopted. Scale bar: 100 μm. **d** The immunoreactivity of the RASD1 expression in GC tissues demonstrated a significantly negative correlation with the relative expression of LINC01446. Spearman’s rank correlation was adopted. **e** The LSD1 knockdown efficiency in GC cells was verified using qRT-PCR. Student’s *t* test was adopted. **f**, **g** MTT and colony formation assays were carried out to determine the proliferation of the RASD1-overexpressed GC cells. Student’s *t* test was adopted. **h** The effects of RASD1 overexpression on the invasion of GC cells were assessed. Scale bar: 120 μm. Student’s *t* test was adopted. **i**, **j** The proliferation of si-LINC01446 and/or si-RASD1-transfected SGC7901 and BGC823 cells was detected using EdU and colony formation assays. Scale bar: 70 μm. Student’s *t* test was adopted. **k** The migration of SGC7901 and BGC823 cells following co-transfection with si-LINC01446 and/or si-RASD1 was detected using transwell assay. Scale bar: 120 μm. Student’s *t* test was adopted. **l** Model of LINC01446 function and mechanisms during GC pathogenesis. Data were shown as mean ± SD, *n* = 3. *P < 0.05. n.s. not significant.

To evaluate whether RASD1 functions as a tumor suppressor in GC, we firstly measured the RASD1 mRNA expression according to the TCGA database. Our data demonstrated that the RASD1 mRNA expression significantly reduced in GC tissues relative to the normal tissues (Fig. 8a, b). Furthermore, immunohistochemical staining assay was also employed to measure the RASD1 expression in 25 matched GC and normal tissues. The representative images were presented in Fig. 8c, d, suggesting that RASD1 was downregulated in GC tissues and was negatively related to the LINC01446 level. Further functional trials demonstrated that RASD1 overexpressing could significantly suppress the proliferation and migration of GC cells (Fig. 8e–h). Next, EdU and colony formation assays were carried out to evaluate whether LINC01446 could regulate the proliferation and migration of GC cells by suppressing RASD1 expression. Our data proved that the co-transfection partly reversed the si-LINC01446-impaired proliferation of SGC7901 and BGC823 cells (Fig. 8i, j). Furthermore, wound-healing and transwell analyses demonstrated that RASD1 knockdown could alleviate the si-LINC01446-impaired migration and invasion of SGC7901 and BGC823 cells (Figs. S4a–e and 8k). Taken together, our data indicated that LINC01446 might promote the proliferation and metastasis of GC cells through partly decreasing RASD1 expression.

## Discussion

GC has always been a severe health threat worldwide especially in the highly endemic region, China^21,22^. Therefore, there is an urgent need to discover clinically risk factors and relevant prognostic biomarkers, thereby developing novel precise therapies. Previous studies almost paid attentions to the effects the protein-coding genes exhibited on disease pathogenesis^23^. Recent advances in high-throughput RNA sequencing showed the widespread expressions of lncRNAs in various cancers^24–26^. Massive evidences indicated that lncRNAs, once regarded as ‘junk RNA’, played distinct roles in the occurrence and progression of cancers, but the lncRNAs specifically related to GC are still not clear^27,28^. Therefore, LINC01446 expressions in GC tissues and cells were detected in our study. And the results indicated that the increased LINC01446 facilitated the proliferation, migration, and invasion of GC cells, which is consistent with previous studies showing that LINC01446 functions as an oncogene in glioblastoma^14^. Accordingly, the overexpression of LINC01446 in GC patients was positively associated with advanced TNM stage and distal metastasis. Furthermore, high LINC01446 expression in GC tissues was associated with the poor prognosis and could be an independent prognostic indicator. These results indicated that LINC01446 might serve as a clinically significant prognostic biomarker for GC and might play an important role in GC progression. So, there is an urgent need to investigate the action mechanism of LINC01446 facilitating the proliferation and metastasis of GC.

In glioblastoma, it is demonstrated that LINC01446 could promote cell proliferation and migration by competing with miRNAs and acting as ceRNAs^14^. In our study, LSD1 was found to be an RNA binding protein to LINC01446 and directly interacted with LINC00460, thereby affecting the proliferation, migration, and invasion of GC cells. Furthermore, it was also found that LSD1 expression markedly enhanced in GC tissues relative to the normal tissues, and LSD1 knockdown obviously impaired the proliferation and migration of GC cells. LSD1 is a member of the flavin-dependent LSD/KDM1 demethylase protein family, and is reported as a transcription repressor that can demethylate H3K4me1/me2^29–32^. To explore the related signals and underlying targets that participate in the regulation of LINC01446-LSD1 axis in GC on an unbiased basis, we conducted high-throughput RNA sequencing following the knockdown of LINC01446 in SGC7901 cells. Further GO and GSEA analyses indicated that the gene expression profiles were primarily associated with proliferation and metastasis. Among these altered genes, we found that RASD1 was markedly upregulated after LINC01446 or LSD1 knockdown. Mechanistic investigations showed that LINC01446 suppressed the transcription of RASD1 by the LSD1-mediated H3K4 demethylation, which suggests that RASD1 is a downstream target gene of LINC01446-LSD1 axis.

RASD1 belonged to the Ras family is originally regarded as a dexamethasone-inducible gene, and its product is a receptor-independent activator of G-protein signal^33,34^. Although there are still some controversies regarding the roles RASD1 played in carcinogenesis, many studies revealed that RASD1 could serve as a tumor suppressor in diverse malignancies^35–38^. For instance, Gao et al. reported that the overexpression of RASD1 in glioma cells possibly played inhibitory roles in the migration and invasion of tumor cells through inactivating AKT/mTOR signal^39^. Furthermore, Govindan et al. found that restoring RASD1 expression in three different cancer cells played a significant anti-growth role^19^. In our current work, RASD1 was obviously downregulated in GC tissues. Functional studies demonstrated that RASD1 could also suppress the proliferation and migration of GC cells. Interestingly, our rescue experiments illustrated that LINC01446 facilitated the proliferation and metastasis of GC partly depending on the suppress of RASD1.

In conclusions, we identified a novel GC-related lncRNA, LINC01446, and firstly demonstrated that LINC01446 could promote the proliferation and metastasis of GC through the epigenetic knockdown of RASD1 via binding to LSD1 (Fig. 8l). In addition, LINC01446 might serve as an underlying prognostic biomarker for GC. These results will provide insights into the development of novel therapies for GC.

## Materials and methods

### Ethics

The experimental protocols for the collection and application of human samples were approved by the Ethics Committee of Zhongshan Hospital, Xiamen University (Xiamen, China). All subjects provided written informed consent for surgery. All animal trials were carried out based on the national and international guidelines and approved by the Animal Care and Use Institutional Review Board of Xiamen University.

### Collection of human samples

After surgery, 100 GC and normal gastric epithelial tissues were immediately harvested from the GC patients at Zhongshan Hospital, Xiamen University and Second Affiliated Hospital, Nanjing Medical University (Nanjing, China). The clinicopathological information about all subjects were acquired according to the patient records, which were listed in Tables 1 and 2.

### Quantitative real-time PCR (qRT-PCR)

In brief, total RNA was firstly extracted from GC cells with TRIzol (Invitrogen). Next, cDNA was synthesized with 1 μg total RNA based on the protocols of kit (Takara). Finally, GAPDH was employed as a negative control to determine the relative mRNA level of target genes in Quant Studio 6 Flex system (Applied Biosystems). All of the primer sequences were listed in Table S1.

### Cell culture and transfection

A normal human gastric epithelium cell line (GES-1) and five GC cell lines including SGC7901, BGC823, MGC803, MKN45, and AGS without mycoplasma were provided by Chinese Academy of Sciences (Shanghai, China). SGC7901, BGC823, and MGC803 cells were grown in RPMI-1640 (Gibco) medium; SGC7901, AGS, and GES-1 cells were incubated in DMEM (Gibco) containing 10% fetal bovine serum (FBS; Gibco), 100 U/ml penicillin and 100 mg/ml streptomycin (Invitrogen) at 37 °C with 5% CO_2_.

Lipofectamine 2000 (Invitrogen) was used to transfect si-LINC01446, si-LSD1, si-RASD1, and negative control siRNA (si-NC). The transfections of pcDNA-LINC01446, pcDNA-RASD1, and empty vectors were performed by using X-tremeGENE HP DNA (Roche). After 24 or 48 h of transfections, the cells were harvested. All of the above siRNA sequences were listed in Table S3.

### Cell proliferation and migration assays

MTT method was firstly used to detect cell viability thereby assessing the cell proliferation. In brief, the transfected SGC7901 and BGC823 cells (3000 cells/well) with si-LINC01446 or pcDNA-LINC01446 were seeded in 96-well plates. Next, cell viability was detected every 24 h based on MTT kit protocols. For colony formation assay, 400 cells were inoculated into sixwell plates and cultured at 37 °C for 14 days. Then, the colonies were fixed with methanol and stained with hematoxylin. For EdU assay, a 5-ethynyl-2-deoxyuridine detection kit (Ribobio) was employed, and DAPI was employed to label the nuclei of GC cells. Finally, the EdU-positive cells were calculated in five randomly selected fields per well under a fluorescent microscopy. All trials were conducted in triplicate.

Wound-healing assay was carried out to measure cell migration. In brief, cells were firstly inoculated in sixwell plates. After 24 h of culture with serum-free medium, the monolayer cells were linearly scraped to generate an artificial wound. For the transwell migration and invasion assays, transwell chambers (Corning) with a membrane pore size of 8 μm were coated without or with Matrigel (BD Biosciences). A total of 5 × 10^4^ or 1 × 10^5^ cells were inoculated in the upper chambers and cultured in the medium supplemented with 1% FBS. Then, the lower chamber was filled using the medium with 10% FBS. After 12 or 24 h of incubation, the cells were fixed, stained, and counted in an inverted microscope. All trials were conducted in triplicate.

### Flow cytometry analysis for apoptosis

After 48 h of transfection with si-LINC01446 or si-NC, SGC7901, and BGC823 cells were harvested. Next, these cells were stained based on the protocols of a FITC Annexin V Apoptosis Detection kit (BD Biosciences), and were subsequently analyzed using flow cytometer (FACScan^®^) and CellQuest software (BD Biosciences). Finally, the cells were assigned into living, dead, early, and late apoptotic cells, and the percentage of early apoptotic cells was calculated using the control cells in each trial.

### In vivo assays for the growth and metastasis of tumors

For the tumorigenicity assay, the stably transfected SGC7901 and BGC823 cells (2 × 10^6^ cells) with sh-LINC01446 or empty vectors were firstly injected into the abdominal cavities of 4 weeks of male BALB/c nude mice (*n* = 6). At the end of this trial, all the mice were sacrificed, and their tumor tissues were weighed and stored for further experiments. In addition, according to the previous report^40^, tumor volume (mm^3^) was calculated by using its longest and shortest diameters.

To evaluate the effects of LINC01446 on tumor metastasis, the stably transfected SGC7901 cells (2 × 10^6^ cells) with sh-LINC01446 or empty vectors were firstly injected into the tail veins of 4 weeks of male BALB/c nude mice (*n* = 6). After 6 weeks, all mice were sacrificed and then their lungs were removed for hematoxylin and eosin (H&E) staining. Finally, the numbers of metastatic tumors in the lungs were observed and calculated in a blinded manner.

### Subcellular fractionation and fluorescent in situ hybridization (FISH)

A PARIS kit (Life Technologies) was used to separate nuclear and cytoplasmic RNAs for qRT-PCR analysis. All of the primer sequences were listed in Table S3. For FISH assay, GC cells, and tissues were firstly fixed with 4% formaldehyde for 15 min after washing with PBS. Then, the fixed cells were incubated in 1% pepsin and dehydrated using 70%, 90%, and 100% ethanol, respectively. Next, the cells were air dried and co-incubated with 40 nM FISH probe diluted in hybridization buffer at 80 °C for 2 min. The hybridization assay was conducted at 55 °C for 2 h, and then the slides were washed and dehydrated. Finally, the slides were air dried and then mounted with Prolong Gold Antifade Reagent containing DAPI for further analysis. RNA FISH probe was synthesized by Bogu Co., Ltd. (Shanghai, China), and its sequences were presented in Table S3.

### RNA immunoprecipitation (RIP) and RNA pull-down assays

RIP was carried out based on the protocols of an EZ-Magna RIP kit (Millipore). In brief, when SGC7901 and BGC823 cells influence reached at 90%, all cells were scraped off and were subsequently lysed using lysis buffer. Next, 100 μL cell extracts were co-incubated with the magnetic beads conjugated with anti-LSD1 or control IgG antibodies (Millipore) at 4 °C for 6 h. Subsequently, the complexes were treated with 0.1% SDS or 0.5 mg/ml proteinase K at 55 °C for 30 min to remove proteins following washing with the washing buffer. Finally, a NanoDrop spectrophotometer (Thermo Scientific) and bioanalyzer (Agilent) were used to measure the concentration and quality of RNA. The purified immunoprecipitated RNA was used for further qRT-PCR analysis.

For the RNA pull-down assay, LINC01446 was transcribed with T7 RNA polymerase (Ambio Life) in vitro, purified using a RNeasy Plus Mini kit (Qiagen), and then treated using RNase-free DNase I (Qiagen). Next, the transcribed LINC01446 was labeled using the Biotin RNA Labeling Mix (Ambio Life). Finally, the RNA pull-down assay was conducted following the protocols of Pierce™ Magnetic RNA-Protein Pull-Down kit (Thermo).

### RNA sequencing

SGC7901 cells were cultured into a sixwell plate and transfected with the siRNA targeting LINC01446 or negative control. After 24 h of transfection, the transfected cells were harvested for RNA extraction, followed by subsequent library construction, and sequencing. RNA sequencing was performed by Novogene, and the raw data could be accessed in the NCBI SRA database (SRA ID: SUB6506100).

### Chromatin immunoprecipitation (ChIP) assay

ChIP assay was conducted based on the protocols of an EZ-ChIP Chromatin Immunoprecipitation kit (Millipore). In brief, sonication was used to proceed the cross-linked chromatin DNA into the 200–500 bp of fragments. Then, the chromatin was immunoprecipitated with the corresponding antibodies. Finally, the isolated DNA was evaluated using qRT-PCR analysis.

### Western blotting and immunocytochemistry (IHC) analysis

Western blotting and IHC analysis were conducted based on the previously reported procedures^40^.

### Statistical analysis

All trials were independently conducted at least three times, with the samples in triplicate. All statistical analyses were carried out with SPSS 22.0 and Prism 5.0 (GraphPad) software. Then, two-tailed Student’s *t* test was used for the assessment of statistical difference between two groups, and Pearson correlation analysis was employed to evaluate the interactions among gene sets. Data were shown as mean ± SD. *P < 0.05 was regarded as significance.

## Supplementary information

Supplementary Table S1

Supplementary Figure Legends

Supplementary Figure S1

Supplementary Figure S2

Supplementary Figure S3

Supplementary Figure S4
