# Supplementary material for: Long intergenic non-protein-coding RNA 01446 facilitates the proliferation and metastasis of gastric cancer cells through interacting with the histone lysine-specific demethylase LSD1
Source: Cell Death Dis. 2020 Jul 10;11(7):522. doi: 10.1038/s41419-020-2729-0 (PMC7351757; doi:10.1038/s41419-020-2729-0)
Supplement: Supplementary file 1 — Supplementary Table S1 [file 41419_2020_2729_MOESM1_ESM.docx]

**Supplementary Table S1**

**Table S1.** The list of primers and siRNA sequences.

| **qRT-PCR primers** | | |
| --- | --- | --- |
| **Gene** | **Forward** | **Reverse** |
| LINC01446 | TTGAAACGCCAGCCAGTT | CCATGTATTTCGCGGTGTTC |
| GAPDH | GAAGAGAGAGACCCTCACGCTG | ACTGTGAGGAGGGGAGATTCAGT |
| LSD1 | AGCGTCATGGTCTTATCAA | GAAATGTGGCAACTCGTC |
| RASD1 | CGACTCGGAGCTGAGTATCC | GCGGATGGAGTAGAACTTGC |
| KLF2 | ACCTGTTGTGTGCATTGGAA | CCTCCTTGCCTGAGACACTT |
| ARNT2 | CCTTTGGGATTGGAACGAG | GAACTGCCCGCTACTTTGTC |
| ARRDC3 | TTTGCCACTTGTCATCGGTA | CAAGATTGTTCCGCCTTTGT |
| BTG2 | CCCTATGAGGTGTCCTACCG | CTGGAGACTGCCATCACGTA |
| DUSP2 | CCTGTGGAGGACAACCAGAT | CCCCATGAAACTGAAGTTGG |
| FOSB | CCCGTTGTTAACCCTTCGTA | CTCTCCCCCATGTGTTTGTT |
| HIC2 | TCTGTGGCAAAATGTTCACG | ACAGCTGGCACTCGTAAGGT |
| CDKN1A | AAGTCAGTTCCTTGTGGAGCC | GGTTCTGACGGACATCCCCA |
| **Primer used for qCHIP analysis Sequences** | | |
| RASD1 E1 region | GGGTGGTATGGCACTCACAG | GTCACTGCTCCTCAAGGCAA |
| RASD1 E2 region | ACATACCTCAGATGGGGGCA | GCAGCTCGACCAACTATCCA |
| **FISH probe** | | |
| LINC01446 | TTCTAATTAAAGTTTCTTCTTGAGTCTTCCATGATTTGAAAGTGT | CAGCAGTTATCCGACAGGCT |
| **siRNA sequences** |  |  |
| si-LINC01446 1# | CCUUGGAGGACUGACUAGGAGCACA |  |
| si-LINC01446 2# | UGUGCUCCUAGUCAGUCCUCCAAGG |  |
| si-LINC01446 3# | GCCUGUUCUGGGCGUCCCGUUGAAA |  |
| si-LSD1 1# | CCGGAUGACUUCUCAAGAATT |  |
| si-LSD1 2# | GCCACCCAGAGAUAUUACUTT |  |
| si-RASD1 1# | CCAAGAACUGCUAUCGCAUGGUCAU |  |
| si-RASD1 2# | GACACCAAGUCUUGCCUCAAGAACA |  |
| si-NC | UUCUCCGAACGUGUCACGUTT |  |
