# Supplementary material for: Long intergenic non-protein-coding RNA 01446 facilitates the proliferation and metastasis of gastric cancer cells through interacting with the histone lysine-specific demethylase LSD1
Source: Cell Death Dis. 2020 Jul 10;11(7):522. doi: 10.1038/s41419-020-2729-0 (PMC7351757; doi:10.1038/s41419-020-2729-0)
Supplement: Supplementary file 2 — Supplementary Figure Legends [file 41419_2020_2729_MOESM2_ESM.docx]

**Supplementary Figures**

**Fig. S1** (a) Left: PCR products from the 5'RACE procedure was shown by agarose gel electrophoresis. Right: Nucleotide sequence of the full-length human LINC01446 RNA in SGC7901. (b) LINC01446 expression profile in normal tissues was obtained from UCSC Genome Browser database. (c) Relative expression of LINC01446 in 3 subtype gastric cancer tissues compared with normal tissue was analyzed by using TCGA data.

**Fig. S2** (a) Representative images (left) and quantification (right) for EdU immunofluorescence staining in the pcDNA-LINC01446- and Empty vector-transfected MGC803 cells. Scale bar: 70μm. (b) Apoptosis in the pcDNA-LINC01446- and Empty vector-transfected MGC803 cells was determined using flow cytometry. (c) TUNEL staining was conducted to measure the cell apoptosis after LINC01446 knockdown. Scale bar: 130μm. (d and e) The migration and invasion of the pcDNA-LINC01446- and Empty vector-transfected MGC803 cells was investigated using wound-healing assay and transwell assays. Scale bar: 120μm. (Data were shown as mean ± SD, n=3. Student’s t-test, **P* < 0.05. n.s., not significant).

**Fig. S3** GSEA showed that the proliferation-and metastasis-related biological functions were enriched in responses to high LINC01446 expression in SGC7901 cells based on the RNA-seq analysis after LINC01446 knockdown.

**Fig. S4** (a-c) The migration and invasion of GC cells following co-transfection with si-LINC01446 and/or si-RASD1 was detected using wound-healing assay and transwell assay. Scale bar: 120μm. (Data were shown as mean ± SD, n=3. Student’s t-test, **P* < 0.05. n.s., not significant).

**Table S1.** The list of primers and siRNA sequences.
