# Supplementary figures and images for: Long intergenic non-protein-coding RNA 01446 facilitates the proliferation and metastasis of gastric cancer cells through interacting with the histone lysine-specific demethylase LSD1
Source: Cell Death Dis. 2020 Jul 10;11(7):522. doi: 10.1038/s41419-020-2729-0 (PMC7351757; doi:10.1038/s41419-020-2729-0)

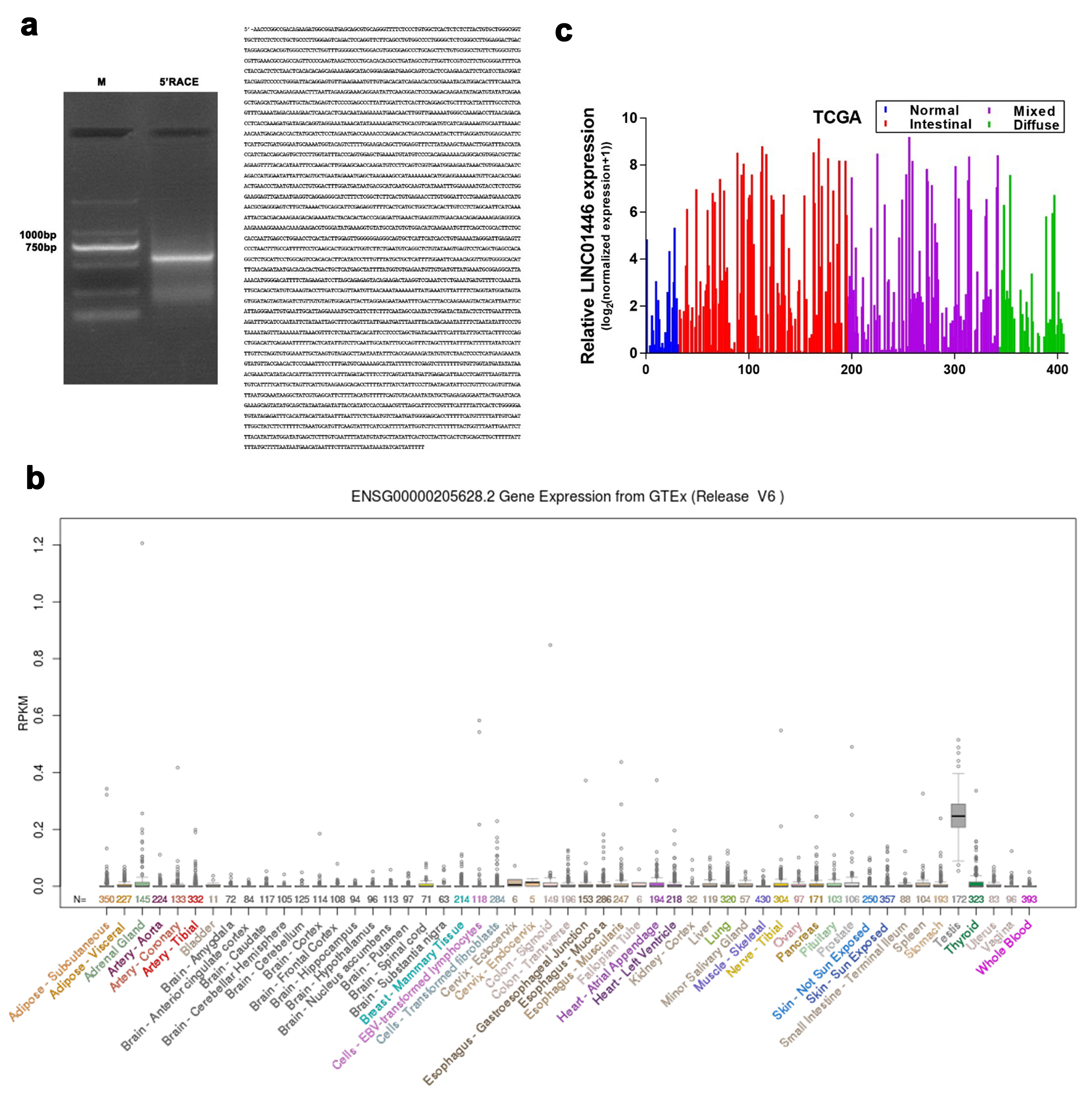

Supplement: Supplementary file 3 — Supplementary Figure S1 [file 41419_2020_2729_MOESM3_ESM.png]

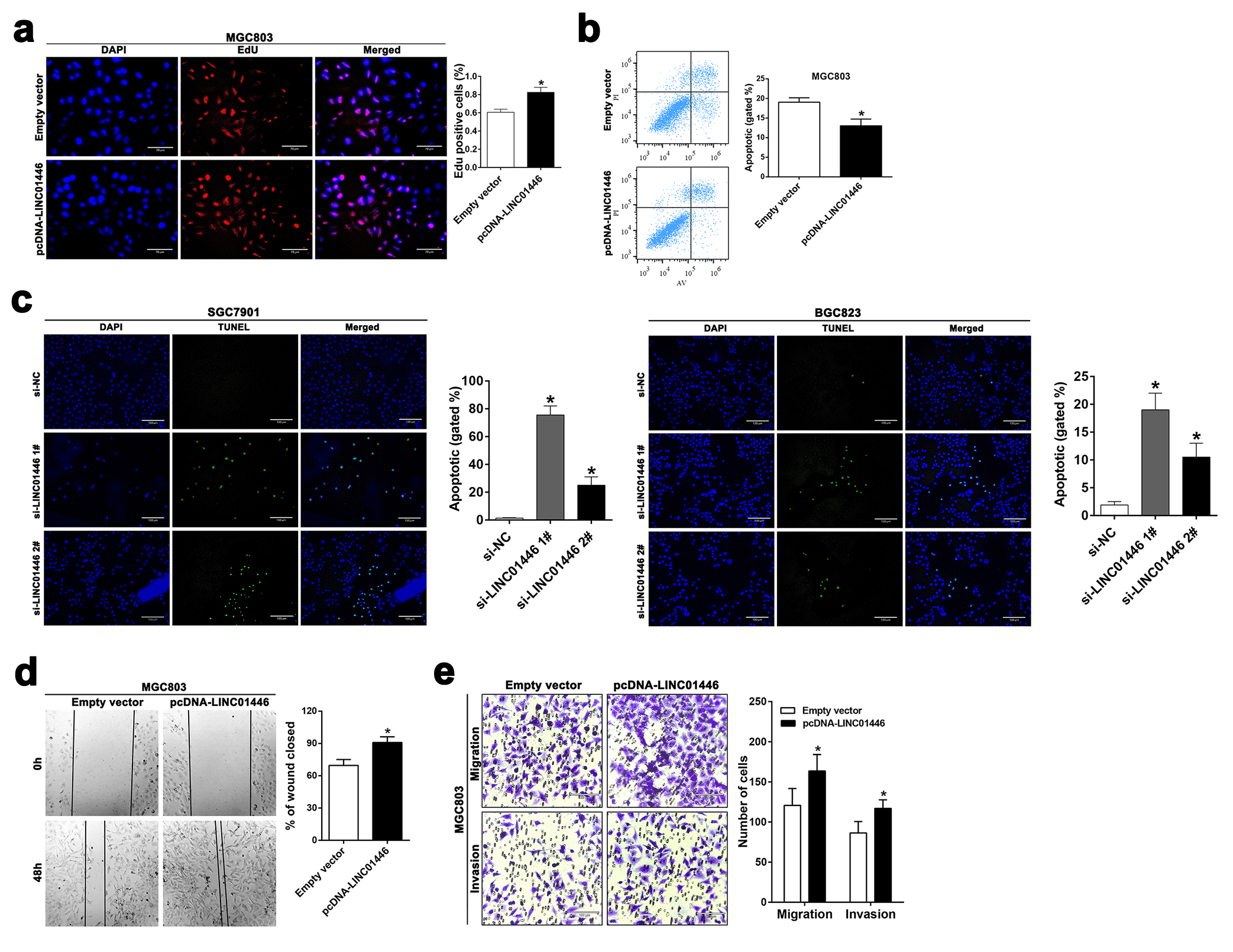

Supplement: Supplementary file 4 — Supplementary Figure S2 [file 41419_2020_2729_MOESM4_ESM.png]

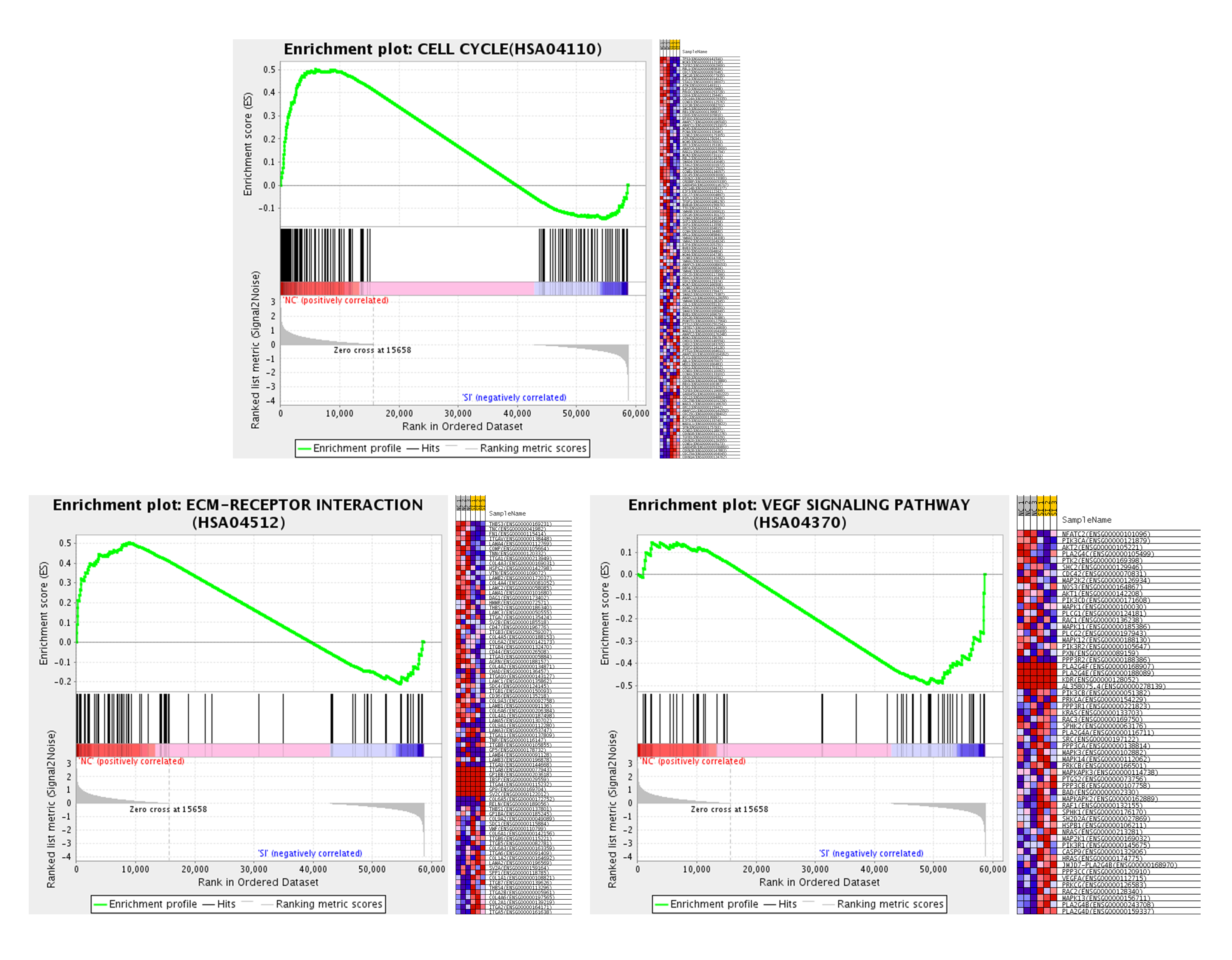

Supplement: Supplementary file 5 — Supplementary Figure S3 [file 41419_2020_2729_MOESM5_ESM.png]

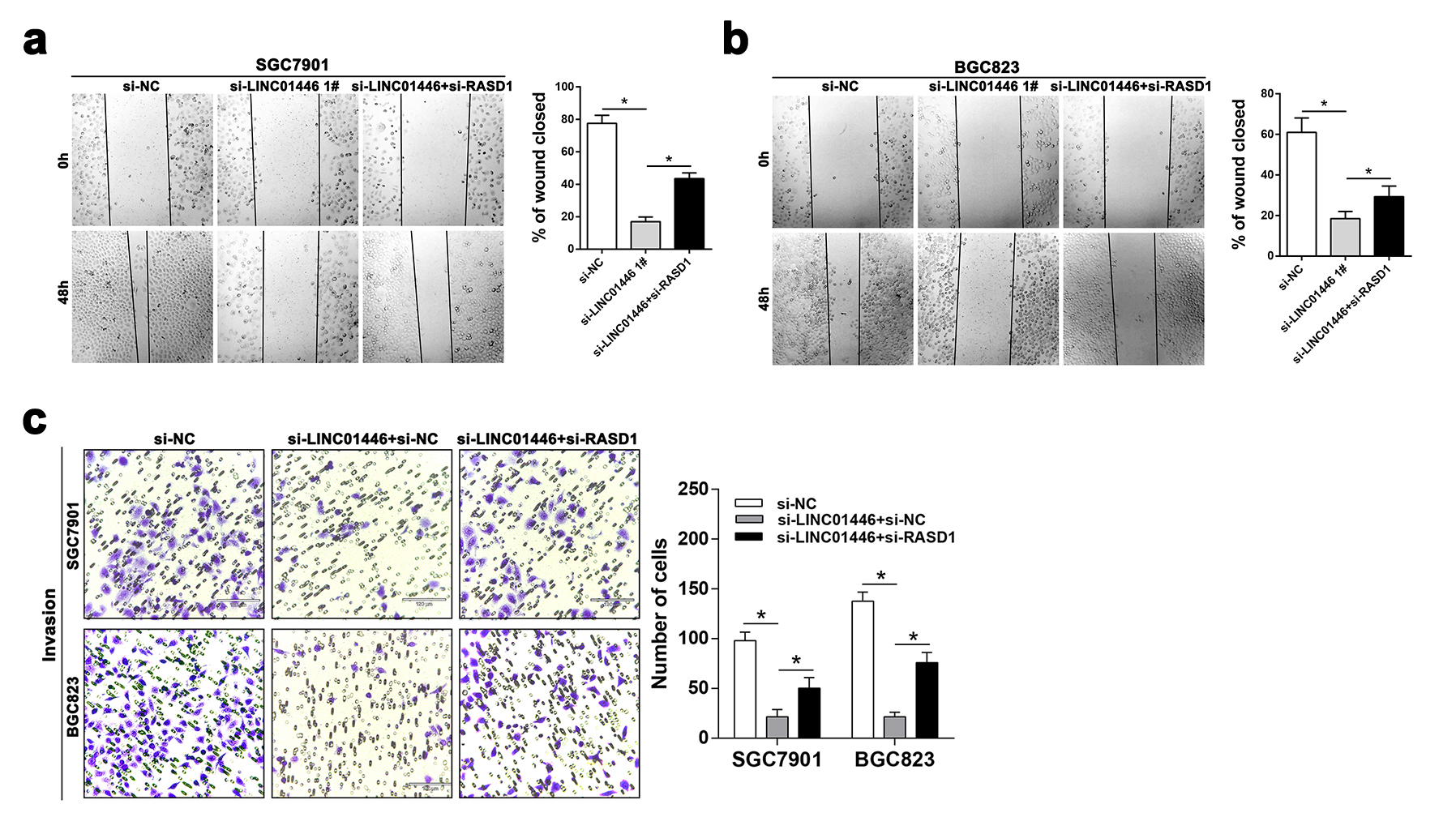

Supplement: Supplementary file 6 — Supplementary Figure S4 [file 41419_2020_2729_MOESM6_ESM.png]
